# Mitochondria-targeted Probes for Imaging Protein Sulfenylation

**DOI:** 10.1038/s41598-018-24493-x

**Published:** 2018-04-27

**Authors:** Reetta J. Holmila, Stephen A. Vance, Xiaofei Chen, Hanzhi Wu, Kirtikar Shukla, Manish S. Bharadwaj, Jade Mims, Zack Wary, Glen Marrs, Ravi Singh, Anthony J. Molina, Leslie B. Poole, S. Bruce King, Cristina M. Furdui

**Affiliations:** 1 Department of Internal Medicine, Section on Molecular Medicine, Wake Forest University Health Sciences, Winston-Salem, NC 27157 USA; 2 Department of Chemistry, Wake Forest University, Winston-Salem, NC 27109 USA; 3 Department of Internal Medicine, Section on Gerontology and Geriatric Medicine, Wake Forest University Health Sciences, Winston-Salem, NC 27157 USA; 4 Department of Biology, Wake Forest University, Winston-Salem, NC 27109 USA; 5 Department of Cancer Biology, Wake Forest University Health Sciences, Winston-Salem, NC 27157 USA; 6 Department of Biochemistry, Wake Forest University Health Sciences, Winston-Salem, NC 27157 USA

## Abstract

Mitochondrial reactive oxygen species (ROS) are essential regulators of cellular signaling, metabolism and epigenetics underlying the pathophysiology of numerous diseases. Despite the critical function of redox regulation in mitochondria, currently there are limited methods available to monitor protein oxidation in this key subcellular organelle. Here, we describe compounds for imaging sulfenylated proteins in mitochondria: DCP-NEt_2_-Coumarin (DCP-NEt_2_C) and rhodamine-based DCP-Rho1. Side-by-side comparison studies are presented on the reactivity of DCP-NEt_2_C and DCP-Rho1 with a model protein sulfenic acid (AhpC-SOH) and mitochondrial localization to identify optimized experimental conditions for labeling and visualization of protein sulfenylation that would be independent of mitochondria membrane potential and would not impact mitochondrial function. These probes are applied to image mitochondrial protein sulfenylation under conditions of serum starvation and in a cell culture model of lung cancer exposed to ionizing radiation and silver nanoparticles, agents serving dual functions as environmental stressors and cancer therapeutics.

## Introduction

Protein oxidation in cells can be caused by reactive oxygen species (ROS), such as hydrogen peroxide (H_2_O_2_), generated by normal metabolism, damaging agents or pathological conditions, or can be a part of normal metabolic processes and signaling. Protein sulfenic acids (-SOH) are formed when cysteine thiols react with H_2_O_2_ and these can be further oxidized to form sulfinic or sulfonic acids, condense with other sulfenic acids to form thiosulfinates, condense with sidechain -NH_2_ or backbone -NH- in proteins to produce sulfenylamides, react with thiols to yield disulfides, or with H_2_S to generate per/polysulfides^1^. Protein sulfenic acids are also formed indirectly when cysteine thiols react with HOSCN or HOX (X-Cl, -Br, -I) to produce -SSCN or -SX species^2^, which then hydrolyze to sulfenic acids^3^. The high reactivity of sulfenic acids creates challenges for their detection, but as the first step of various oxidative processes, these species are an attractive target for studying protein oxidation^4–8^.

Mitochondria generate ATP through oxidative phosphorylation and regulate cellular metabolism. Mitochondria are involved in the maintenance of cell viability and function, but also play a central role in the regulation of apoptosis. The mitochondrial respiratory chain is a major source of ROS within cells. The main type of ROS generated in mitochondria is superoxide (O_2_^•−^) that is rapidly dismutated to H_2_O_2_ and oxygen by the mitochondrial manganese-dependent superoxide dismutase (SOD2, MnSOD)^9,10^. Mitochondrial damage and dysfunction along with increased mitochondrial ROS are implicated in a range of human diseases including aging^11,12^, cancer^13^ and neurodegenerative diseases, such as Parkinson’s disease^14,15^.

Changes in the thiol redox state of mitochondrial proteins are significant in a number of cellular processes^16,17^ and whereas several reagents are already available for selective manipulation and detection of ROS in mitochondria^18–21^, corresponding methods for direct detection of their protein targets remain limited^22–24^. Most information regarding the biological roles of protein sulfenylation comes from studies using nucleophilic 1,3-dicarbonyl-based probes, such as dimedone that irreversibly alkylates these species^25–30^. Chemical probes in this class have detectable and/or enrichment tags such as a fluorescent dyes or biotin to enable analysis of protein sulfenylation by imaging, western blotting or mass spectrometry^1,25,28,31^. With few exceptions, the application of these probes for labeling sulfenylated proteins in mitochondria has not been investigated^23,24^. While we considered a number of methods for targeting protein sulfenylation probes to mitochondria such as the use of triphenyl phosphine (TPP) and peptides like Szeto-Schiller (SS) and mitochondria-penetrating peptides (MPP)^32,33^, these mitochondria-targeting tags lack fluorescence signals or robust antibodies for visualization of labeled proteins by imaging.

As some studies have demonstrated labeling of mitochondria with fluorescent coumarin and rhodamine compounds^34,35^, we focused our initial efforts on the synthesis of a new protein sulfenylation probe, DCP-NEt_2_-Coumarin (DCP-NEt_2_C), and compared its properties with DCP-Rho1, a rhodamine-labeled probe synthesized earlier^29^, but which has not been characterized with respect to its mitochondria targeting properties. The studies presented here include evaluation of: 1) the reactivity of DCP-NEt_2_C and DCP-Rho1 with a model protein sulfenic acid (AhpC-SOH) relative to dimedone (which has the same reactive core as “DCP”, the abbreviation used for the 3-(2,4-dioxocyclohexyl)propyl group), 2) mitochondrial localization, 3) dependence of mitochondrial localization on mitochondrial membrane potential, and 4) impact of labeling sulfenylated proteins with DCP-NEt_2_C and DCP-Rho1 on mitochondrial respiration. Finally, the application of these probes to measure protein sulfenylation induced by serum starvation as a representative model for pathophysiological processes, and by ionizing radiation and silver nanoparticles (AgNPs) in lung cancer cells as pathological models, are presented as case studies. The data show that while both compounds label sulfenylated proteins in mitochondria, DCP-NEt_2_C is better suited for biological imaging applications as it does not display mitochondrial toxicity or dependence of uptake on mitochondrial membrane potential over the relevant concentration range.

## Results

### Synthesis and Characterization of Mitochondria-targeted Protein Sulfenylation Probes

**Figure 1:**
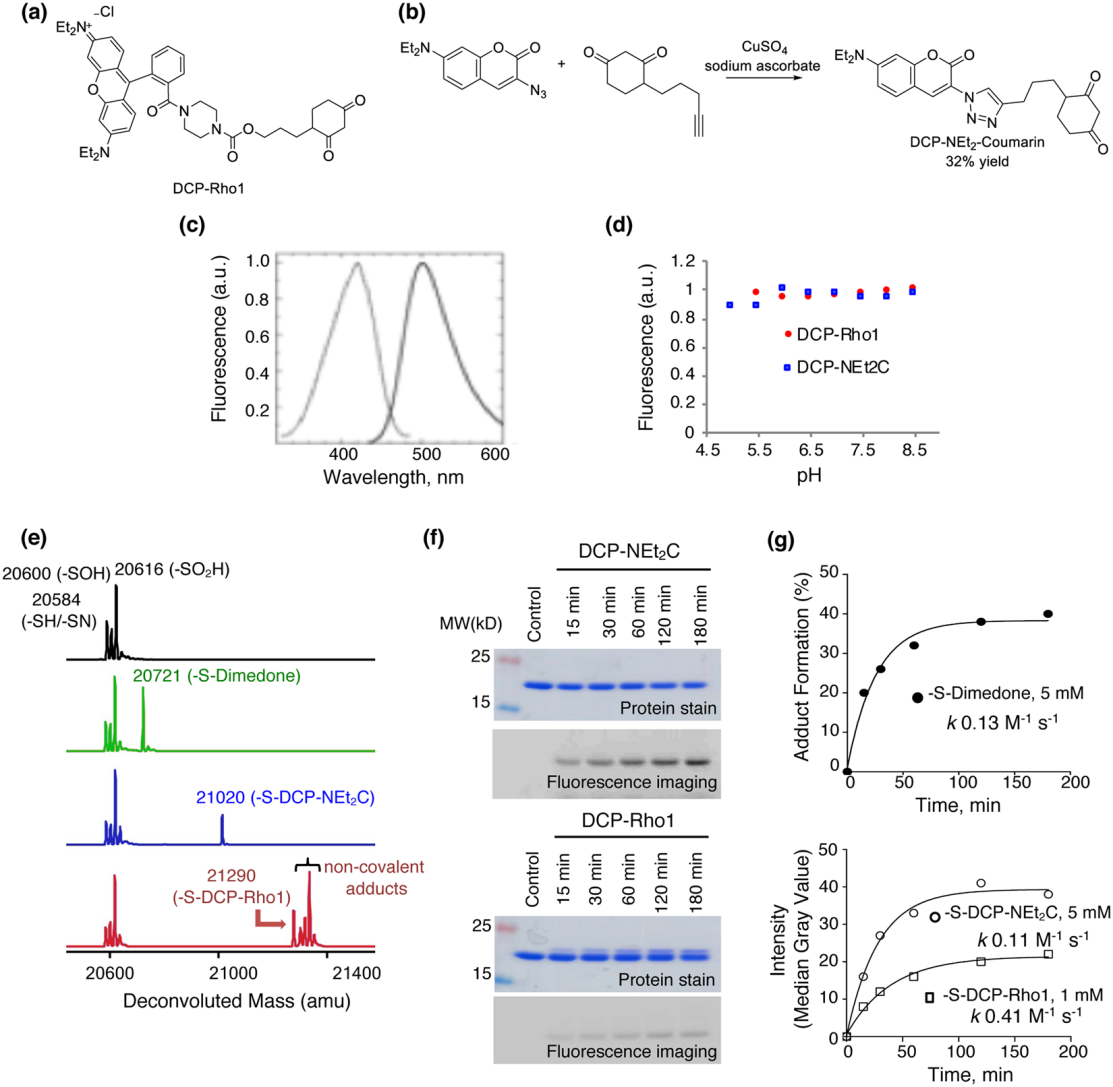
Characterization of DCP-NEt_2_C and DCP-Rho1 fluorescence and kinetic properties. (**a**) Chemical structure of DCP-Rho1. (**b**) DCP-NEt_2_C synthesis scheme and structure. (**c**) Excitation and emission spectra of DCP-NEt_2_C. Dotted line shows excitation spectrum taken with emission at λ_max_ 499 nm; solid line line shows emission spectrum taken with excitation at λ_max_ 421 nm. (**d**) Analysis of DCP-NEt_2_C (blue) and DCP-Rho1 (red) dependence of fluorescence signal on pH. (**e**) Deconvoluted mass spectra of oxidized C165A AhpC (black), and its 60 min reaction with dimedone (green), DCP-NEt_2_C (blue), and DCP-Rho1 (red). (**f**) SDS-PAGE fluorescence imaging analysis of oxidized C165A AhpC reaction with DCP-NEt_2_C and DCP-Rho1. (**g**) Kinetic profiles and reaction rates of oxidized C165A AhpC reaction with dimedone (black circle), DCP-NEt_2_C (open circle), and DCP-Rho1 (open square). The data were fit using single exponential kinetics and the second order rate constants were calculated by dividing the rate with the respective probe concentration.

We have previously reported the synthesis of a rhodamine-tagged protein sulfenylation reagent, DCP-Rho1^29^ (λ_ex, max_ at 560 nm, λ_em, max_ at 581 nm) (Fig. 1a). For the synthesis of DCP-NEt_2_C, we applied the widely used copper-catalyzed Click reaction to covalently link the previously described DCP-alkyne^36^ with the known diethyl amine-containing coumarin azide^37^ to generate DCP-NEt_2_C in 32% yield (Fig. 1b). The synthetic procedure and characterization of DCP-NEt_2_C can be found in the *Methods* section. The analysis of its fluorescence properties showed maximum excitation wavelength (λ_ex, max_) at 421 nm and maximum emission wavelength (λ_em, max_) at 499 nm (Fig. 1c).

As shifts in mitochondrial pH can occur with changes in mitochondrial respiration^38^, it is important for both the reactivity of probes and fluorescence signal to be independent of pH to correctly assign changes in fluorescence to changes in protein sulfenylation. The independence of dimedone/DCP reactivity on pH was reported earlier^28^. Here we monitored the fluorescence signal of DCP-NEt_2_C and DCP-Rho1 within the pH range of 5 to 8.5 and found the fluorescence of both compounds to be independent of pH within the mitochondria-relevant pH range of 6.8–7.7 (Fig. 1d)^38^. Next, we tested the reactivity of the DCP-NEt_2_C and DCP-Rho1 with a protein sulfenic acid model relative to dimedone. We have used the *S. typhimurium* peroxiredoxin AhpC where the resolving Cys165 was mutated to alanine. The C165A AhpC mutant allows for formation of sulfenic acid at the peroxidatic Cys46 upon oxidation, enabling kinetic studies^26,27,29,39,40^. The reaction rates of the mitochondria-targeted probes with C165A AhpC-SOH were determined by monitoring the appearance of alkylated protein modifications after the addition of probes using mass spectrometry (dimedone) or gel-based fluorescence for DCP-NEt_2_C and DCP-Rho1 probes (Fig. 1e,f). DCP-NEt_2_C displayed comparable reactivity with dimedone towards C165A AhpC-SOH (-S-Dimedone: *k* = 0.13 M^−1^ s^−1^, -S-DCP-NEt_2_C: *k* = 0.11 M^−1^ s^−1^), while DCP-Rho1 showed approximately 3-fold higher second order rate constant (*k* = 0.41 M^−1^ s^−1^) (Fig. 1g). To further confirm that the adduct formation was driven by the DCP moiety, control experiments were performed using rhodamine B and the NEt_2_C-N_3_ precursor for DCP-NEt_2_C (*Supplemental Information*, Fig. S1). While no adduct was noted with rhodamine B when this was reacted with either the reduced or oxidized C165A AhpC (*Supplemental Information*, Fig. S1a–c), the formation of two new species was observed when NEt_2_C-N_3_ was reacted with reduced C165A AhpC. These were identified as the products of azide reaction with the reduced thiol in AhpC (–NH-S-AhpC) and the resolved protein species after the loss of sulfur (−32 Da) (*Supplemental Information*, Fig. S1a–c). The finding of the -NH-S- adduct was expected and is supported by previous literature demonstrating the reaction of azides with thiols^41^. The mechanisms leading to the loss of sulfur are unclear, but a potential explanation could be reactions in gas phase during mass spectrometry analysis leading to loss of -S-NH-NEt_2_C. The lack of an adduct matching the addition of 256 Da delta mass (AhpC-S-NEt_2_C-N_3_) demonstrates lack of reactivity of this coumarin moiety with the AhpC-SH. Lack of coumarin and rhodamine moieties’ reaction with general protein thiols is also substantiated by flow cytometry data showing lack of protein labeling when NEt_2_C-NH_2_ (intermediate in synthesis of DCP-NEt_2_C)^37^ and rhodamine B were added to cells (*Supplemental Information*, Fig. S1d).

### Subcellular Localization of DCP-NEt2C and DCP-Rho1 Fluorescence Signal

**Figure 2:**
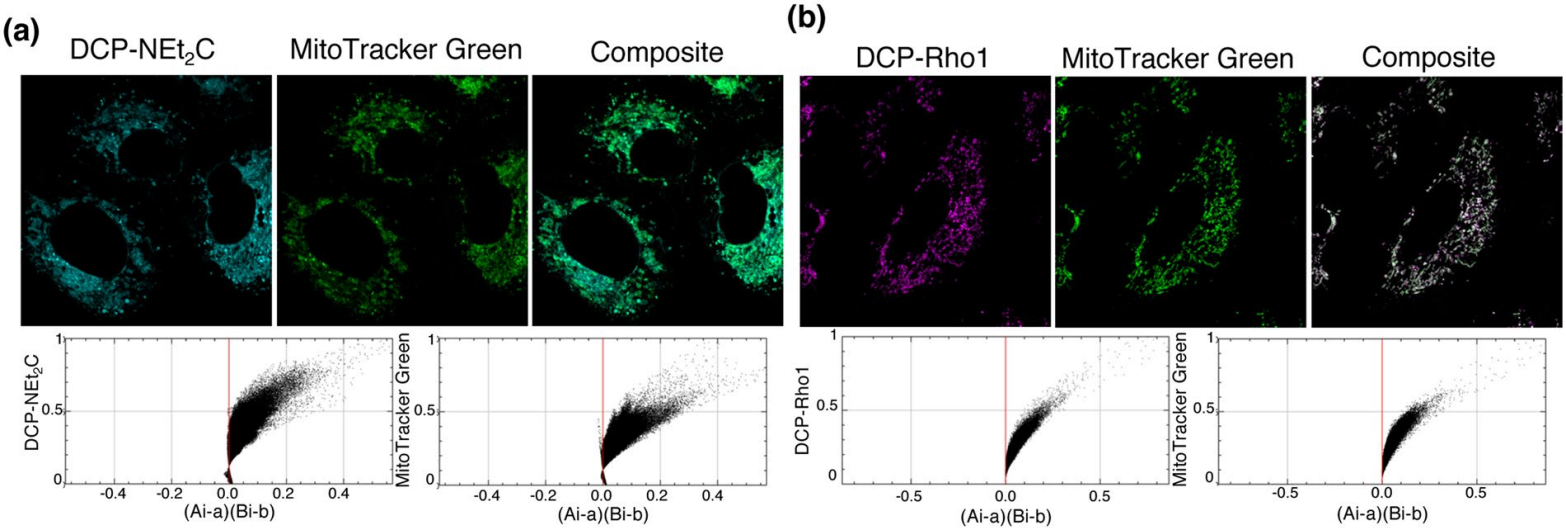
Subcellular localization of the probes in live cells. (**a**) A549 cells were treated with DCP-NEt_2_C (cyan color) and imaged live; MitoTracker Green (green color) was used as mitochondrial marker. ICA-plots show colocalization analysis (ICA: intensity correlation analysis). (**b**) A549 cells were treated with DCP-Rho1 (magenta color) and imaged live, MitoTracker Green (green color) was used as mitochondrial marker. ICA-plots show colocalization analysis.

The uptake of DCP-NEt_2_C and DCP-Rho1 into cells and analysis of subcellular localization were performed using confocal imaging of live and fixed cells to distinguish between free and protein bound probe, and subcellular fractionation with fluorescence SDS-PAGE analysis to further validate the results. Confocal imaging analysis of DCP-NEt_2_C and DCP-Rho1 subcellular distribution in live cells (Fig. 2a,b, respectively) shows colocalization with the mitochondria marker MitoTracker Green. The colocalization analysis with Li’s method^42^ showed an almost perfect colocalization of DCP-Rho1 with MitoTracker Green (Li’s Intensity correlation coefficient ICQ: 0.47; ICQ values range from −0.5 to 0.5 with 0.5 representing perfect colocalization). DCP-NEt_2_C also showed strong colocalization with MitoTracker Green, but with slightly lower ICQ (0.38).

In a separate experiment, after 30 min exposure to the probes, the cells were fixed with cold methanol and the unreacted probes were washed away as described in the *Methods* section. While the signal intensity was decreased by removing the free unreacted probes, the results of colocalization imaging analysis were consistent with live cell imaging, although some loss of colocalization is noted primarily for DCP-Rho1 (ICQ for DCP-Rho1 colocalization with TOMM20: 0.28, and for DCP-NEt_2_C colocalization with TOMM20: 0.35) (*Supplemental Information*, Fig. S2a,b). We also performed experiments with a biotin-tagged DCP probe (DCP-Bio1) imaged using Streptavidin tagged with Alexa Fluor 594 to determine if DCP itself may target the probes to the mitochondria (*Supplemental Information*, Fig. S2c). Under basal conditions, there was a weak overall fluorescence signal that was confounded with the background of cellular biotinylated proteins. Cell treatment with the pro-oxidant *t*-butyl hydroperoxide (tBHP), however, increased labeling with DCP-Bio1 in all cell compartments demonstrating that it is the NEt_2_C and Rho1 groups that drive localization of DCP-NEt_2_C and DCP-Rho1 to the mitochondria. Data in *Supplemental Information*, Fig. S1d point as well to lack of reaction of the coumarin and rhodamine moieties with proteins or other cellular macromolecules. Notably, we also attempted to generate the gem-dimethyl analogs of DCP-NEt_2_C and DCP-Rho1 as additional negative controls using published methods^24^. This was not successful likely due to the presence of numerous reactive groups in DCP-NEt_2_C, in particular, interfering with this reaction. An alternative synthetic approach of clicking a gem-dimethyl DCP-alkyne with the azide of the coumarin probe may be warranted for generation of such a control for future studies. Subcellular fractionation of cells treated with the DCP-NEt_2_C and DCP-Rho1 probes into nuclear, cytosolic, mitochondrial and membrane fractions followed by fluorescence SDS-PAGE analysis also revealed enriched labeling of sulfenylated proteins in the mitochondrial fraction, further supporting the localization and covalent reaction of both probes in the mitochondria (*Supplemental Information*, Fig. S3).

**Figure 3:**
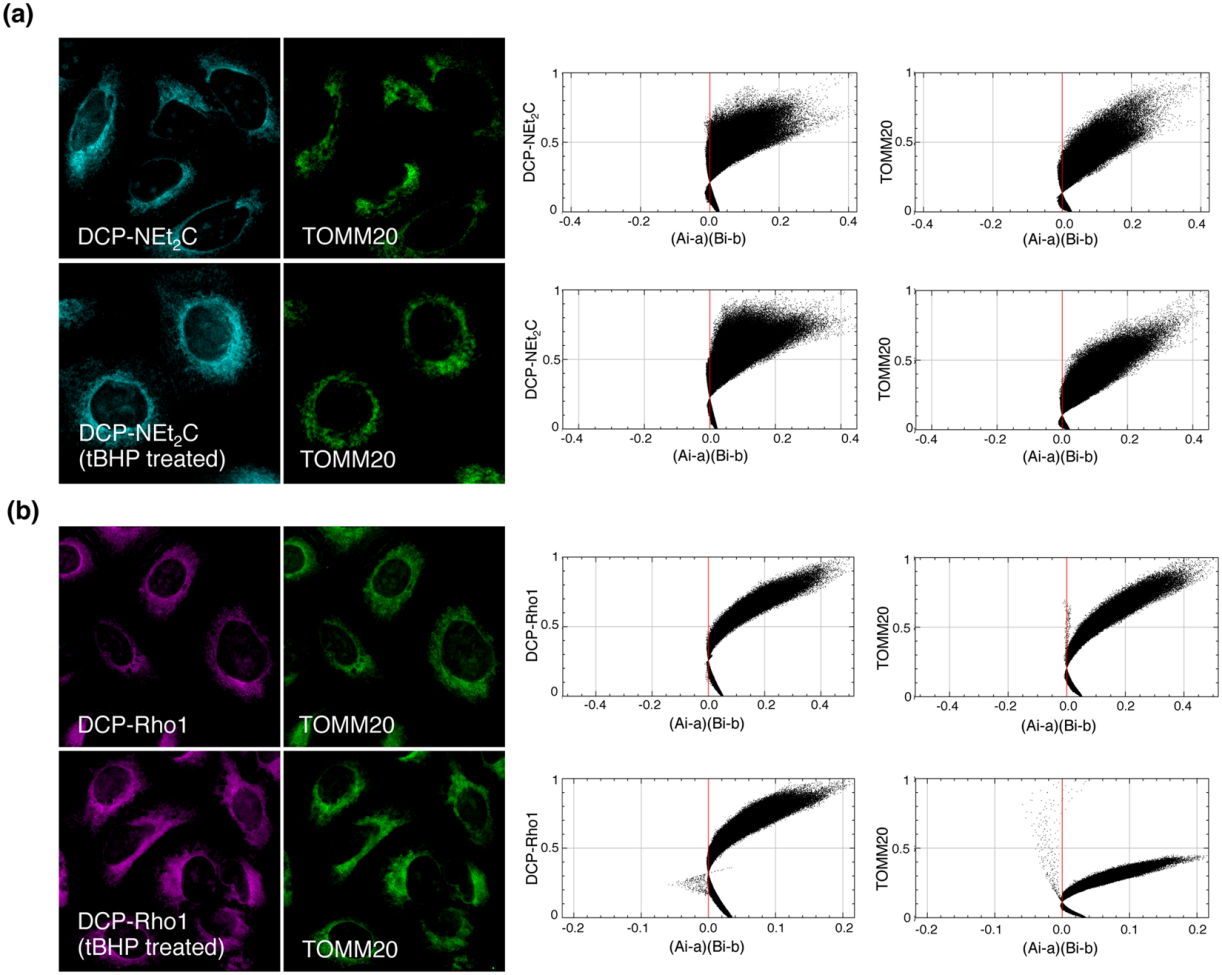
Labeling of mitochondria sulfenylated proteins during fixation. (**a**) A549 cells were fixed with methanol containing DCP-NEt_2_C (cyan color); TOMM20 (green color) was used as mitochondrial marker. ICA plots show colocalization analysis. (**b**) A549 cells were fixed with methanol containing DCP-Rho1 (magenta color); TOMM20 (green color) was used as mitochondrial marker. ICA-plots show colocalization analysis.

An additional study was performed where the sulfenylated proteins were labeled with DCP-NEt_2_C and DCP-Rho1 during the methanol fixation. This seemed to better conserve the colocalization of DCP-Rho1 with the mitochondrial marker TOMM20 (ICQ: 0.45), whereas not much change was seen for DCP-NEt_2_C (ICQ: 0.33) compared with the experiment where the cells were fixed after addition of probes to live cells (Fig. 3a for DCP-NEt_2_C; Fig. 3b for DCP-Rho1 data). The extent of colocalization was not impacted by increasing general oxidative stress in cells with tBHP treatment before labeling the sulfenylated proteins during the fixation (ICQ for DCP-NEt_2_C 0.34 and for DCP-Rho1 0.44, Fig. 3a,b).

### Cellular Uptake and Effects of Mitochondrial Membrane Potential on DCP-NEt_2_C and DCP-Rho1 Subcellular Localization

**Figure 4:**
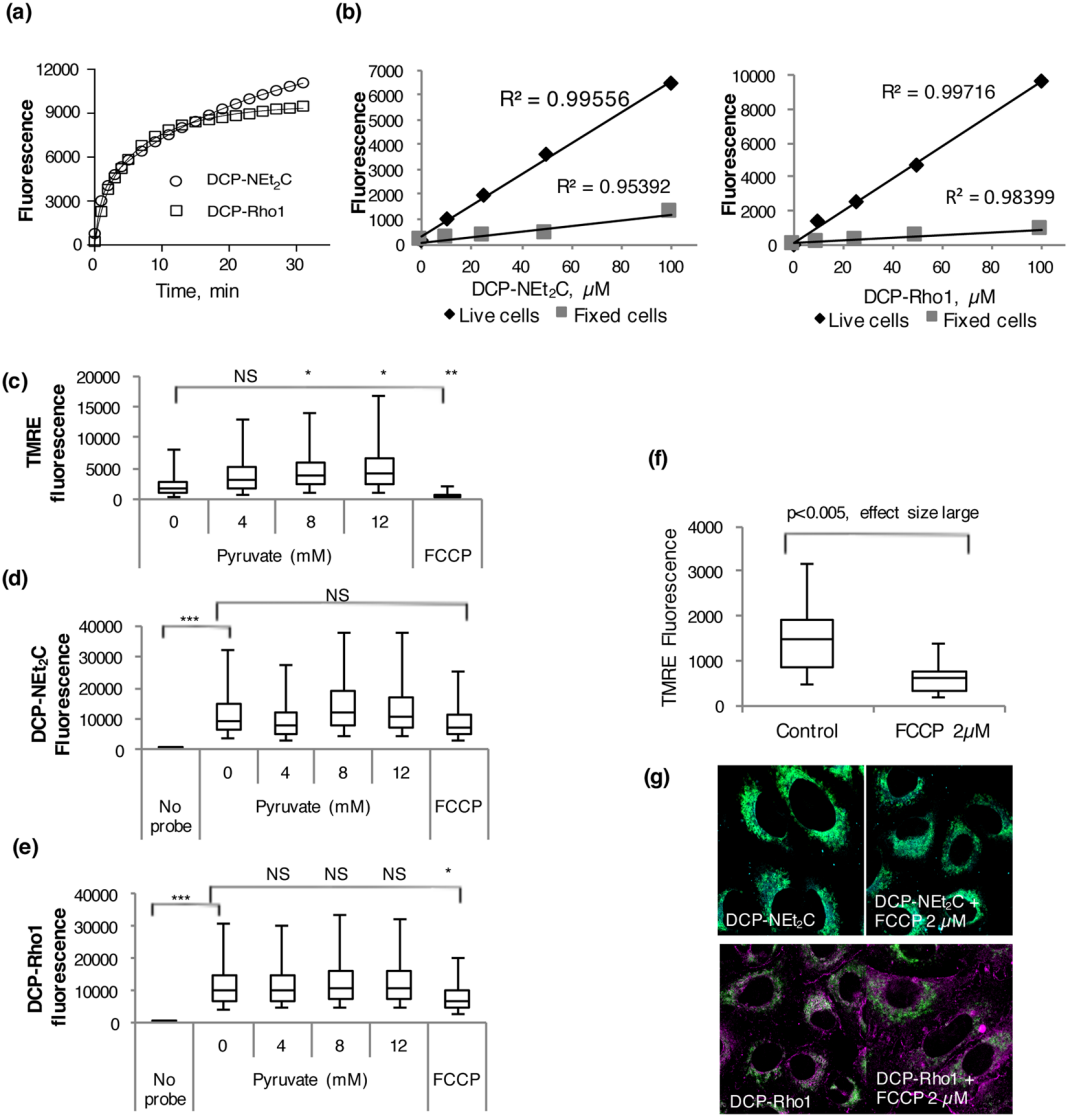
Probe uptake and effect of mitochondrial membrane potential. (**a**) The uptake of DCP-NEt_2_C and DCP-Rho1 into live cells was monitored over time by imaging analysis. The change in fluorescence intensity was fit to a bi-exponential equation to extract the kinetic parameters of uptake into cells. (**b**) The uptake of probes in live and fixed cells measured by flow cytometry was linearly correlated with the probe concentration for both DCP-NEt_2_C and DCP-Rho1, the symbols represent the mean fluorescence for each concentration. (**c**) The effect of pyruvate concentration and FCCP on mitochondrial membrane potential using isolated mitochondria. Compared to 0 mM pyruvate: NS = effect size zero or small; *p < 0.005, effect size medium; **p < 0.005, effect size large. (**d**) The effect of pyruvate concentration and FCCP on DCP-NEt_2_C uptake in isolated mitochondria. Compared to 0 mM pyruvate: NS = effect size small, compared to no probe: ***p < 0.005, effect size very large. (**e**) The effect of pyruvate concentration and FCCP on DCP-Rho1 uptake in isolated mitochondria. Compared to 0 mM pyruvate: NS = effect size small, *p < 0.005, effect size medium, compared to no probe: ***p < 0.005, effect size very large, (**f**) The effect of FCCP on mitochondria membrane potential in live cells using flow cytometry. (**g**) Composite images of the DCP-NEt_2_C probe (cyan color) or DCP-Rho1 (magenta) with mitochondrial marker TOMM20 (green) in methanol fixed A549 cells; the corresponding ICA plots from colocalization analyses are shown in Fig. S4.

The kinetics of DCP-NEt_2_C and DCP-Rho1 uptake was monitored using live cell imaging (Fig. 4a). The profiles were fit to a bi-exponential equation interpreted as differences in the rates of uptake into the cytosol from extracellular space and from cytosol to the mitochondria. The uptake into the cytosol occurs quickly for both probes (*t*_1*/2*_ 0.68 min for DCP-NEt_2_C and 1.4 min for DCP-Rho1) while the enrichment into the mitochondria is faster for DCP-Rho1 (*t*_*1/2*_ 7.4 min) compared with DCP-NEt_2_C (*t*_*1/2*_ 16.6 min). To determine whether the uptake of probes into cells takes place by active transport or diffusion, we monitored next the fluorescence signal at increasing concentrations of DCP-NEt_2_C and DCP-Rho1 in live and fixed cells using flow cytometry. A linear dependence of fluorescence signal on probe concentration was noted in both cases, without any sign of saturation up to 100 μM, pointing to diffusion across cell membranes as the main mechanism of transport into the cells and mitochondria (Fig. 4b).

The relationship between mitochondrial membrane potential and localization of probes along with labeling of sulfenylated proteins is critical to biological applications. Ideally, mitochondrial localization of a probe should not depend on or affect mitochondrial respiration or membrane potential. Thus, we tested first the impact of mitochondrial membrane potential on DCP-NEt_2_C and DCP-Rho1 uptake in isolated mitochondria by analyzing their fluorescence with flow cytometry. Pyruvate was used to increase mitochondrial membrane potential and the mitochondrial decoupler carbonyl cyanide 4-(trifluoromethoxy)phenylhydrazone (FCCP) was used to disrupt the mitochondrial membrane potential. The changes in mitochondrial membrane potential were tested under the same treatment conditions with tetramethyl rhodamine ethyl ester (TMRE) that accumulates in mitochondria depending on the mitochondrial membrane potential^43^. The data as presented in Fig. 4c–e show the uptake of both DCP-NEt_2_C and DCP-Rho1 into isolated mitochondria to be largely independent of shifts in mitochondrial potential with a decrease in the uptake noted only with the FCCP treatment for DCP-Rho1 (which bears a positive charge). While some decrease in fluorescence signal with FCCP treatment is also observed for DCP-NEt_2_C (Fig. 4d), this was not statistically significant.

To further investigate the dependence of mitochondria colocalization of probes on mitochondria membrane potential, we also treated cells with FCCP followed by labeling with DCP-NEt_2_C and DCP-Rho1 and imaging analysis. As in isolated mitochondria, the flow cytometry analysis of TMRE was used to measure mitochondria membrane potential confirming the decrease in mitochondria membrane potential with FCCP treatment (Fig. 4f) similar to data in Fig. 4c. Confocal imaging analysis of DCP-NEt_2_C and DCP-Rho1 colocalization with the mitochondrial marker TOMM20 in fixed cells revealed that the DCP-Rho1 localization to mitochondria was decreased when the mitochondrial membrane potential was disrupted (ICQ: 0.28 without FCCP, 0.22 with FCCP, Fig. 4g and *Supplemental Information* Fig. S4b), whereas the DCP-NEt_2_C localization seemed to be less affected (ICQ: 0.38 without FCCP, 0.35 with FCCP, Fig. 4g and *Supplemental Information* S4a). In live cell imaging studies, a significant drop in the fluorescence signal from DCP-Rho1 was detected after the FCCP treatment, whereas DCP-NEt_2_C was again largely unaffected (*Supplemental Information*, Fig. S5).

### Effects of DCP-NEt_2_C and DCP-Rho1 on Mitochondrial Respiration and Mitochondria Redox State

**Figure 5:**
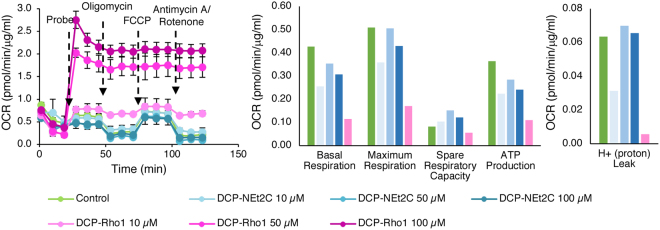
Effect of DCP-NEt_2_C and DCP-Rho1 on mitochondrial respiration. The DCP-NEt_2_C (cyan lines and bars) does not significantly disrupt mitochondrial oxygen consumption rate (OCR) as compared to the control (green line and bars), but DCP-Rho1 (magenta lines and bars) quickly disrupts mitochondrial function in a dose-dependent manner.

Regardless of the experimental design of biological studies, it is critical to identify conditions where the mitochondria-targeted probes do not interfere with mitochondrial function. To investigate the potential effect of the probes on mitochondrial function, we monitored mitochondrial respiration by measuring the oxygen consumption rate (OCR) at increasing probe concentrations. There seemed to be little or no effect of DCP-NEt_2_C on OCR and extracted respirometry parameters (basal respiration, maximum respiration, spare respiratory capacity, proton leak, and ATP production), whereas DCP-Rho1 caused decreased basal respiration even at 10 μM and rapid uncoupling of mitochondria respiration at concentrations above 10 μM (Fig. 5). Consistent with these results, the DCP-Rho1 also increased mitochondrial and cellular oxidative stress, as shown by increase in the hyperoxidation of peroxiredoxins (*Supplemental Information*, Fig. S6). Such an increase was not seen for the DCP-NEt_2_C.

### Response of Mitochondrial Protein Sulfenylation to Oxidative Challenge

**Figure 6:**
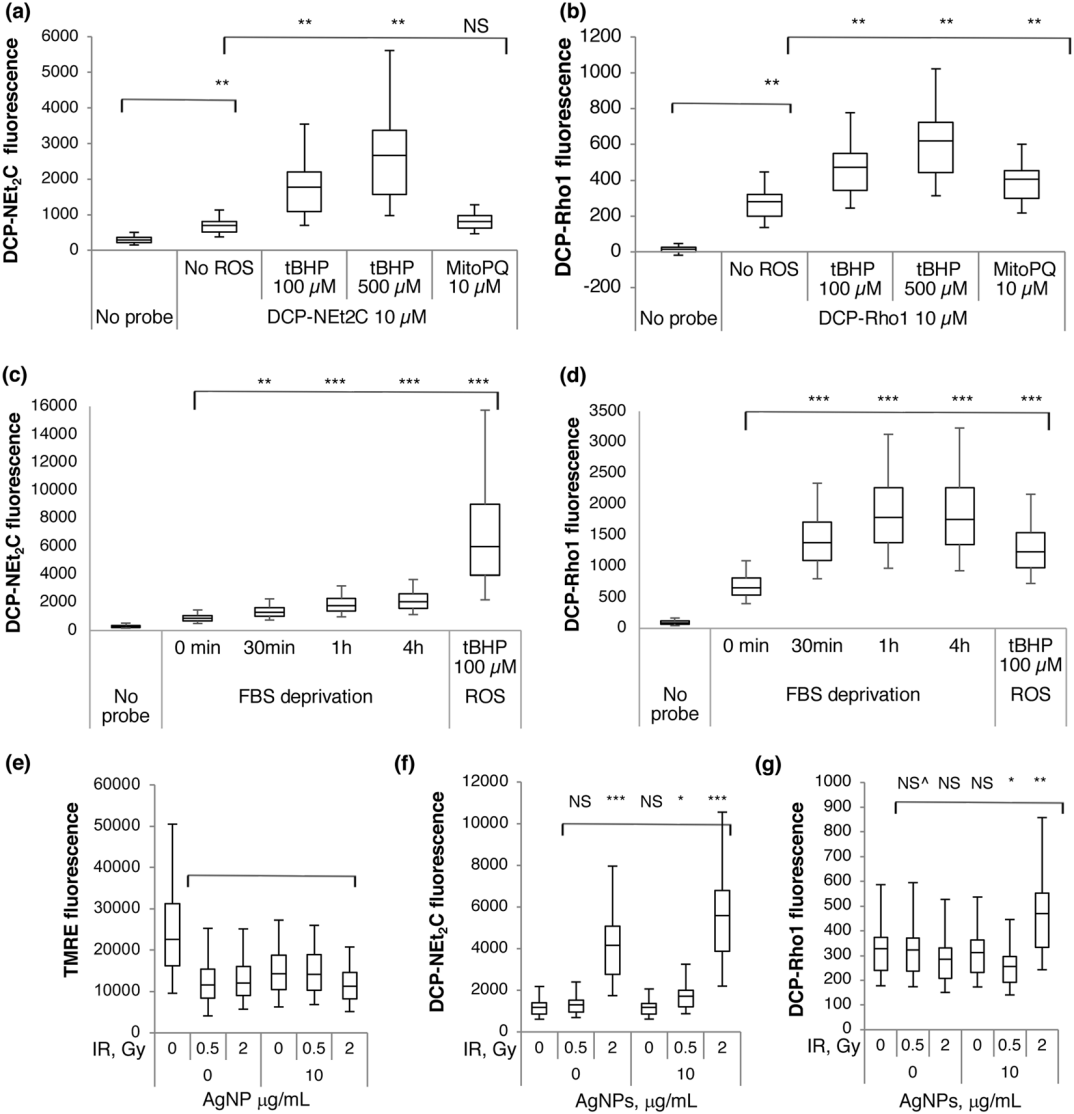
Labeling of sulfenylated proteins in mitochondria under increased oxidative conditions. (**a**) The effect of increased oxidant levels on DCP-NEt_2_C labeling in A549 cells. Compared to no probe or no ROS: NS = effect size small, **p < 0.005, effect size large. (**b**) The effect of increased oxidant levels on DCP-Rho1 labeling in A549 cells. Compared to no probe or no ROS: **p < 0.005, effect size large. (**c**) The effect of fetal bovine serum (FBS) deprivation on DCP-NEt_2_C (50 µM) labeling in A549 cells. Compared to no probe or no ROS: **p < 0.005, effect size large, ***p < 0.005, effect size very large. (**d**) The effect of fetal bovine serum (FBS) deprivation on DCP-Rho1 (10 µM) labeling in A549 cells. Compared to no probe or no ROS: ***p < 0.005, effect size very large. (**e**) Decrease in mitochondrial membrane potential caused by AgNPs and irradiation measured by TMRE: compared to no exposure, **p < 0.0005, effect size large. (**f**) Labeling of sulfenylated protein by DCP-NEt_2_C in cells exposed to AgNPs and irradiation: compared to no exposure, NS p < 0.0005, effect size small, *p < 0.0005, effect size medium, ***p < 0.0005, effect size very large. (**g**) Labeling of sulfenylated protein by DCP-Rho1 in cells exposed to AgNPs and irradiation: compared to no exposure, NS^ p = 0.06, effect size very small, NS p < 0.0005, effect size small, *p < 0.0005, effect size medium, **p < 0.0005, effect size large.

To quantify the correlation of DCP-NEt_2_C and DCP-Rho1 labeled protein sulfenylation with changes in intracellular oxidative state, we treated cells with 100 and 500 μM tBHP, or with 10 μM MitoPQ^20^, to induce increased H_2_O_2_ more broadly and specifically in mitochondria, respectively, and monitored the fluorescence by flow cytometry following treatment with probes. When cells were treated with tBHP there was a clear and dose-dependent increase in the signal from both DCP-NEt_2_C and DCP-Rho1 (Fig. 6a,b). Similarly, the mitochondria-targeted redox cycler MitoPQ increased the fluorescence signal of both probes (Fig. 6a,b).

As a case study for biological applications, we have used DCP-NEt_2_C and DCP-Rho1 to monitor changes in mitochondrial protein sulfenylation with serum starvation (Fig. 6c,d) or increasing doses of ionizing radiation and AgNPs in A549 lung cancer cells (Fig. 6e–g).

#### Serum starvation

Serum starvation induces mitochondrial ROS that can lead to autophagy and apoptosis^44,45^, processes important from both physiological and pathological standpoints^46^. Depriving the A549 cells of fetal bovine serum increased the DCP-NEt_2_C fluorescent signal significantly in a time dependent manner, but the starvation-induced labeling stayed lower compared to the tBHP-induced labeling (Fig. 6c). On the other hand, the serum starvation increased DCP-Rho1 labeling (Fig. 6d) at a level comparable with the labeling induced by tBHP, likely due to the additional oxidative stress induced by DCP-Rho1 itself (*Supplemental Information*, Fig. S6).

#### Ionizing radiation and AgNPs treatment

For these studies, we used spherical AgNPs that possessed a solid core diameter of 5 nm and were stabilized with a dense layer of polyvinylpyrrolidone (PVP), a polymer approved by the US Food and Drug Administration for use in humans. After hydration in water, we characterized the nanoparticle dispersion using dynamic light scattering (DLS). As shown in Fig. S7a and b, the hydrated AgNPs possessed a monomodal size distribution, hydrodynamic diameter of 5–8 nm and a ζ-potential of approximately −35 mV. We also determined the cytotoxicity of AgNPs in the A549 lung cancer cells. AgNPs were found to induce a dose-dependent decrease in A549 cell proliferation with an IC_50_ 28 µg/mL (Fig. S7c). TMRE staining data in Fig. 6e show that both ionizing radiation and AgNPs decreased mitochondrial membrane potential. The irradiation of A549 cells with or without AgNPs increased labeling of sulfenylated proteins in mitochondria by DCP-NEt_2_C in a dose-dependent manner (Fig. 6f), whereas no such dependence with treatment conditions was seen with DCP-Rho1 except for the highest radiation dose combined with AgNPs treatment (Fig. 6g). This could be due to mixed effects of DCP-Rho1 having lower reactivity with sulfenylated proteins compared with DCP-NEt_2_C and a stronger dependence of DCP-Rho1 uptake into the mitochondria on mitochondrial membrane potential.

## Discussion

Mitochondria play key roles in fundamental cell processes, such as energy production, metabolism, cell death, synthesis of heme and iron-sulfur clusters and regulation of calcium, copper, manganese and iron. They are also major sources of ROS in the cell, which is kept in balance by mitochondrial antioxidant systems such as SOD2, Prx3, Prx5, Trx2/TrxR2 and GPx/GR, and others. Thus, there is considerable interest in mitochondria and mitochondrial redox processes under both normal and pathological conditions. Protein thiols play an important role in mitochondrial antioxidant defense and their reversible modifications are part of redox signal transduction and possibly mitochondria-cell communication^47,48^. Detecting changes in mitochondrial redox state related to human pathologies and environmental exposures and identifying specific mitochondria redox-regulated proteins would provide insights on mitochondrial function and could also prove useful as biomarkers of exposure and/or progression to disease.

Here we focused on development of mitochondria-targeted probes for quantifying protein sulfenylation, a key oxidative modification of proteins at cysteine residues^1^. A series of probes targeting these species have been developed over the years, which differ in the mechanism of reaction, reactivity, pH profiles and other parameters providing advantages for desired applications^23,24,26,27,29,39,49^. In this study, we present the development and evaluation of coumarin and rhodamine-linked DCP probes (part of the 1,3-dicarbonyl series structurally similar to dimedone) as potential probes for monitoring protein sulfenylation in mitochondria. Both DCP-NEt_2_C and DCP-Rho1 have fluorescent tags for detection: coumarin in the case of DCP-NEt_2_C that has emission in the blue range, and rhodamine for the DCP-Rho1 with the emission in the red range (Fig. 1). The fluorescence signal of both probes was not dependent on pH and the reactivity with a recombinant sulfenic acid model protein showed the reactivity to be approximately equal or 3-fold higher for DCP-NEt_2_C and DCP-Rho1, respectively, compared with untagged dimedone.

When added to live cells, both probes localized to the mitochondria (Fig. 2). DCP-Rho1 showed an almost complete colocalization with MitoTracker Green, suggesting targeting of the same sub-mitochondrial space by DCP-Rho1 and MitoTracker Green. The time course profiles of DCP-NEt_2_C and DCP-Rho1 uptake monitored by imaging showed faster accumulation of DCP-Rho1 (*t*_*1/2*_ 7.4 min) into the mitochondria compared with DCP-NEt_2_C (*t*_*1/2*_ 16.6 min) (Fig. 4a). As calculated from the second order rate constants shown in Fig. 1, the *t*_*1/2*_ for the reaction of AhpC-SOH with DCP-NEt_2_C and DCP-Rho1 is 21 min and 29 min, respectively, providing the kinetic basis for enriched labeling of mitochondrial sulfenylated proteins. These data, combined with the results of uptake experiments at different probe concentrations (Fig. 4b), colocalization imaging (Fig. 2), and subcellular fractionation results (Fig. S2), show that both probes accumulate and label sulfenylated proteins predominantly in the mitochondria. In the case of DCP-Rho1, the localization is dependent on the mitochondria membrane potential, whereas DCP-NEt_2_C showed lesser dependence (Fig. 4c–g). The signal from the DCP-NEt_2_C and DCP-Rho1 was also linearly dependent on the probe concentration in the 10 to 100 μM range, pointing to passive transport across cell membranes as the main mechanism of uptake into the cells and mitochondria (Fig. 4b). This is consistent with literature on the kinetics of uptake of the rhodamine dyes showing active transport to be dominant only at concentrations <2 μM^50,51^. The localization of probes to the mitochondria was retained after fixation of cells and washing to remove the unbound excess probe (Fig. 3), which reduced the fluorescence signal from the probes by ~5-fold. Removing the excess probe is important as the signal from protein-bound probe should be distinguished from the free probe accumulated in mitochondria. Fixation of cells also allows simultaneous detection of other cellular structures using immunohistochemistry.

Mitochondrial respirometry analysis showed minimum impact on basal respiration and other extracted parameters by DCP-NEt_2_C at all concentrations tested, while DCP-Rho1 drastically increased the mitochondrial oxygen consumption rate at concentrations above 10 μM (Fig. 5). Based on the cumulative data presented, the DCP-Rho1 should only be used at concentrations below 10 μM when added to live cells. Both the probe concentration and the exposure time should be calibrated for each cell type investigated and for both probes to ensure limitation of artifacts due to probe effects on mitochondrial function. To avoid the interference of the probes with cellular and mitochondrial function, the probes could also be used to label sulfenylated proteins during methanol fixation, but some of the mitochondrial specificity may be lost as shown here (Fig. 3).

When the cells were exposed to oxidative stress, either general or mitochondria specific, before and during the treatment with the probes, there was a clear dose-dependent increase in protein labeling by DCP-NEt_2_C and DCP-Rho1 (Fig. 6a,b). As an example of biological application of these probes, we analyzed the effect of serum starvation and treatment with AgNPs and ionizing radiation, separate or combined, on mitochondrial protein sulfenylation in A549 cells. Serum starvation can induce autophagy to promote cell survival by recycling cellular components to support metabolism^46,52^. Even though the mechanisms are still not clearly understood, it is clear that the mitochondrial ROS play a role in these processes^53–55^. Here we showed that depriving the A549 of serum increased mitochondrial protein sulfenylation significantly and in a time-dependent manner. The increase in fluorescence was even higher with the DCP-Rho1, comparable with the tBHP stimulation, further pointing out the compromising effects of DCP-Rho1 on mitochondria.

It is generally accepted that the toxicity of AgNPs is due primarily to the release of silver cation (Ag^+^) from the nanoparticles. AgNPs may function as a “Trojan Horse”^56^ and carry silver metal across cell membranes to then generate a sustained flux or bolus release Ag^+^ from intracellular depots and damage mitochondria and other sensitive intracellular targets^57^. Ionizing radiation exerts its effects mainly through DNA damage but also through oxidation of proteins, lipids, and other macromolecules^58^. Indeed, we could detect using DCP-NEt_2_C a clear increase in protein sulfenylation in the presence of AgNPs, which augments the signal induced by ionizing radiation alone (Fig. 6f). With DCP-Rho1, the increase in signal was seen only for the highest dose of radiation with AgNPs (Fig. 6g), probably due to the decrease in mitochondrial potential induced by the same treatments, stressing again the need for testing and validating the probes for a specific experimental setup.

In summary, we present here the development and evaluation of two fluorescent mitochondria-targeted probes for analyzing protein sulfenylation compatible with imaging and flow cytometry. Both probes localize to the mitochondria and respond to increases in intracellular or extracellular oxidative state, with significant differences residing in their dependence on mitochondrial membrane potential and impact on mitochondrial function. In the context of the case studies included here, DCP-NEt_2_C displayed superior properties compared with DCP-Rho1. These should prove useful tools for studying the redox processes in mitochondria and their role in response to environmental exposures and disease progression.

## Methods

### Chemical Synthesis

#### General

Reagents were obtained from commercial sources and used without additional purification. Reaction solvents such as dichloromethane, acetonitrile, and ethyl acetate were dried and distilled over calcium hydride. Extraction, silica, and preparative reverse phase chromatography solvents were technical grade. Analytical TLC was performed on silica gel plates, and visualization was accomplished with UV light. ^1^H NMR spectra were recorded on Bruker Avance DPX-300 and DRX-500 instruments at 300.13 and 500.13 MHz, respectively. ^13^C NMR spectra were recorded on the described instruments operating at 75.48 and 125.76 MHz, respectively. Low-resolution mass spectra were obtained using an Agilent Technologies 1100 LC/MSD ion trap mass spectrometer equipped with an atmospheric pressure electrospray ionization source and was operated in positive ion mode unless otherwise noted.

#### 4-(3-(1-(7-(diethylamino)-2-oxo-2H-chromen-3-yl)-1H-1,2,3-triazol-4-yl)propyl)cyclohexane-1,3-dione (DCP-NEt_2_-Coumarin)

A solution of sodium ascorbate (12 mg, 0.06 mmol) and copper sulfate (4.5 mg, 0.028 mmol) in water (0.5 mL) was added to a solution of *N*-ethyl coumarin azide (60 mg, 0.23 mmol) and DCP alkyne (42 mg, 0.23 mmol) in acetone/ethanol/water (4.5/9/4.5 mL). This mixture was stirred for 24 h at room temperature, the solvent was removed under vacuum and the crude product purified over silica (methanol, 1 to 8% gradient and chloroform) to give the final product in 32% yield.

^*1*^*H-NMR (*3*00 MHz, CDCl*_*3*_*, TMS):* δ 8.36 (s, 1 H), 8.32 (s, 1 H), 7.41 (d, *J* = 6.0, 1 H), 6.68 (dd, *J*_1_ = 6.0 Hz, *J*_2_ = 3.0 Hz, 1 H), 6.56(d, *J* = 3.0, 1 H), 3.45 (m, 6 H), 2.83 (m, 2 H), 2.70 (m, 1 H), 2.58 (m, 2 H), 2.18 (m, 1 H), 1.99 (m, 1 H), 1.85 (m, 2 H), 1.57 (m, 2 H), 1.25 (t, *J* = 6.0 Hz, 6 H).

^13^*C-NMR (75 MHz, CDCl*_3_*, TMS):* δ. 204.55, 204.01, 157.07, 155.75, 151.51, 147.34, 134.5, 130.03, 129.94, 121.86, 117.07, 110.05, 107.12, 107.68, 97.02, 77.23, 58.29, 49.13, 45.00, 39.67, 28.62, 26.70, 25.60, 24.44, 12.43.

*LRMS-ESI*^+^ *(m/z):* [M + H]^+^ predicted 437.2, found 437.2.

### Dependence of DCP-Rho1 and DCP-NEt_2_C Fluorescence Signal on pH

Stock solutions of 200 mM DCP-Rho1 and DCP-NEt_2_C were prepared and diluted to 5 μM final concentration in different pH buffers. The buffer composition was 10 mM monobasic sodium phosphate, 10 mM sodium citrate, 10 mM boric acid, and 1 mM EDTA. The pH was adjusted from pH 5 in 0.5 unit increments up to pH 8.5 with NaOH. The spectra were recorded using Tecan Infinite M1000 Pro with 5 nm bandwidths for both excitation and emission wavelengths using 96-well Costar clear, flat bottom plates, measured from the bottom.

### Kinetic Studies of C165A AhpC-SOH Reaction with Dimedone, DCP-Rho1, and DCP-NEt_2_C

The C165A mutant of *Salmonella typhimurium* AhpC was overexpressed in *E. coli* and purified as described in previous work^59^. 1,4-Dithiothreitol (DTT) (10 mM, ThermoFisher Scientific) was used to reduce C165A AhpC for 60 min at room temperature (RT), and then DTT was removed by passing the solution through a Bio-Gel P6 (Bio-Rad) spin column which was equilibrated with ammonium bicarbonate (50 mM, pH 7.5). Protein concentration was determined using ε_280_ of 24,300 M^−1^cm^−1^. After incubation with 1–1.2 equivalents of H_2_O_2_ (Sigma-Aldrich) for 40 s at room temperature in ammonium bicarbonate buffer (50 mM, pH 7.5, Sigma-Aldrich), the sulfenic acid species of C165A AhpC (C165A AhpC-SOH) was generated. Unreacted H_2_O_2_ was removed by passing the reaction mixture through a Bio-Gel P6 spin column equilibrated with ammonium bicarbonate (50 mM, pH 7.5). The C165A AhpC-SOH (50 μM, also containing -SN and -SO_2/3_H species) was incubated with 5 mM dimedone, 5 mM DCP-NEt_2_C, or 1 mM DCP-Rho1 probes at room temperature and analyzed at different time points (15 min, 30 min, 60 min, 120 min and 180 min) by ESI-TOF MS (Electrospray Ionization Time-of-Flight Mass Spectrometry) on an Agilent 6120 MSD-TOF system. The analysis was performed in positive ion mode with settings as follows: capillary voltage: 3.5 kV, nebulizer gas pressure: 30 psig, drying gas flow: 5 L/min, fragmentor voltage: 200 V, skimmer voltage: 65 V, and gas temperature: 325 °C. Agilent MassHunter Workstation software v B.02.00 was used for average and deconvolution of mass spectra. For the control experiments, both reduced and oxidized C165A AhpC were reacted for 60 min with 2 or 5 mM NEt_2_C-N_3_ and rhodamine B (Sigma R6626) as indicated in Fig. S1 (*Supplemental Information*) prior to analysis by ESI-TOF MS. The presence of reduced thiol was tested with the thiol-selective blocker methylsulfonyl benzothiazole (MSBT, 5 mM)^60^.

### Cell Culture and Treatment

A549 cells were obtained from the American Type Culture Collection. Cells were cultured at 37 °C under 5% CO_2_, in Ham’s F-12K (Kaighn’s) Medium (Gibco) supplemented with 10% fetal bovine serum (Sigma-Aldrich), 100 U/mL penicillin and 100 μg/mL streptomycin (Gibco). The cells were treated with the probes, DCP-NEt_2_C or DCP-Rho1, at indicated concentrations (typically 10 μM DCP-Rho1, 10 μM or 50 μM DCP-NEt_2_C and 100 μM DCP-Bio1) in F-12K medium without serum for 30 min at 37 °C under 5% CO_2_ and washed three times with DPBS (Lonza) before the next step. For live cell imaging, the cells were also treated with 10 nM MitoTracker Green (Thermo Fisher) simultaneously with the probes. For the ROS induction, the cells were first treated for 10 min with 100 μM or 500 μM tert-butyl hydroperoxide (tBHP) (Sigma)^61^ or 10 μM MitoPQ (Cayman Chemical), after which the DCP-NEt_2_C or DCP-Rho1 probes were added as above. For manipulating the mitochondrial membrane potential, the cells were first incubated with 2 μM FCCP (Cayman Chemical) for 10 min after which the DCP-NEt_2_C or DCP-Rho1 probes were added as above. In control experiments, 10 nM TMRE was incubated for 15 min to confirm membrane depolarization by FCCP treatment.

In the starvation experiments the cells were cultured in the F-12K medium without fetal bovine serum for different time periods as indicated with probe addition to the starvation media for the last 30 min of incubation. In the case of 30 min starvation, the probes were added at the time when cell culture media was changed to starvation media. In the control sample (0 min), the probes were added in the complete media containing 10% fetal bovine serum for 30 min. For the experiments with AgNPs and ionizing radiation, the cells were first exposed to AgNP (10 μg/mL) in Ham’s F-12K medium with fetal bovine serum for 24 h. The ionizing radiation was applied with a 444 TBq 12,000 Ci self-shielded ^137^Cs (Cesium) irradiator with indicated doses and the cells were labeled with probes as before one hour after the irradiation.

### Imaging Analysis

After cell treatment, the imaging was done immediately in live cells or alternatively the cells were fixed for 5 min with cold (−20 °C) methanol. After fixation the cells were washed two times with DPBS and blocked with 1% BSA in PBS-T (0.1% Triton x-100 (Sigma) in DPBS) for 1 h at room temperature. After blocking, the slides were incubated with anti-TOMM20 antibody tagged with Alex Fluor 488 (Abcam, ab205486, dilution 1:1000) for one hour at room temperature for visualization of the mitochondria. After antibody incubation the cells were washed 3 × 5 min with DPBS and mounted with VectaShield mounting mediuam (VectorLabs). Imaging was done using the Zeiss 880 confocal with Airyscan mode (laser excitation 405 nm for DCP-NEt_2_C, 488 nm for TOMM20 and MitoTracker Green and 560 nm for DCP-Rho1). The images were processed using Fiji ImageJ^62^. Briefly, the threshold was automatically defined using Otsu-method and brightness adjusted, using the same values for any comparative analyses. The colocalization was analyzed with Li’s method using ImageJ JaCoP plugin^42,63^.

### Mitochondrial Respirometry Analysis and Redox State

The effect of probes on the mitochondrial function was measured using Seahorse Mito Stress Test following the manufacturer’s protocol. Briefly, the day before the planned studies, 6 × 10^4^ A549 cells/well were plated on a Seahorse plate and incubated overnight. The next day the assay media and compounds (to final concentrations of 1 μM for Oligomycin (Fisher Scientific), 1 μM for FCCP (Cayman Chemical) and 1 μM Antimycin (Abcam)/Rotenone (Sigma-Aldrich)) were prepared according to Seahorse protocols. The cells were washed twice with assay media and incubated at 37 °C without CO_2_ for 1 h after which the analysis was run with Seahorse XF 24 (Agilent Technologies). After the Seahorse analysis, the cells were lysed with modified RIPA buffer (50 mM Tris-HCl, pH 7.4; 1% NP40; 0.25% Sodium deoxycholate; 15 mM NaCl; 1 mM EDTA; 1 mM NaF; supplemented with Roche protease and phosphatase inhibitor tablets and 200 U/mL catalase) and protein concentration was measured with BCA (Thermo Scientific) for data normalization. The data were analyzed with Wave software (Agilent Technologies). Basal respiration was calculated as the difference in the averaged OCR values of the first 3 time points after the probe addition and the last 3 time points after the addition of antimycin A/rotenone. Maximum respiration was calculated as the difference in the averaged OCR values of the 3 time points recorded before and after the addition of antimycin A/rotenone. ATP production was calculated as the difference in the averaged OCR values for the 3 time points recorded before and after the addition of oligomycin. Proton leak was calculated as the difference in the averaged OCR values recorded at the 3 time points after addition of oligomycin and the ones after antimycin A/rotenone addition. Spare capacity was calculated as the difference between maximal respiration and basal respiration. Mitochondrial redox state was evaluated using peroxiredoxin hyperoxidation as redox biomarker. A549 cells were treated with 50 μM DCP-NEt_2_C or 10 μM DCP-Rho1 for 30 min at 37 °C under 5% CO2, washed twice with DPBS, and lysed in RIPA buffer (150 mM NaCl, 50 mM Tris pH7.4, 1% NP-40, 0.5% sodium deoxycholate, 1% SDS) supplemented with 200 U/ml catalase and protease and phosphatase inhibitors. The crude lysate was incubated on ice for 30 min before clarified by centrifugation at 13,000 rpm at 4 °C for 10 min. Protein concentration was measured using the BCA assay. Equal amounts of samples were loaded on a 12% SDS-PAGE gel, transferred onto a nitrocellulose membrane, and analyzed by Western blotting using anti-PRX-SO_2/3_ (Abcam ab16830, dilution 1:2000) and anti-PRX3 (Santa Cruz sc-59611, dilution 1:1000) antibodies. The chemiluminescence signal was generated using Amersham ECL Prime (GE Healthcare, RPN2232). The blot was stained with Pierce Reversible Protein Stain (ThermoFisher 24580) as controls for equal protein loading.

### Flow Cytometry

After cell treatment, the cells were detached from the plates with trypsin and resuspended for live cell analysis or fixed for 5 min with cold (−20 °C) methanol. Fixed cells were washed with DPBS (Lonza) before resuspending in DPBS for analysis. About 10,000 cells were analyzed for each condition. The flow cytometry experiments were conducted with a BD Fortessa Cell Analyzer (BD Biosciences) and data were analyzed using the FCS Express v3 software (De Novo Software). The Students’ t-test was used to compare the mean fluorescence values of different conditions and in addition the effect size was calculated using formula d = (M_1_-M_2_)/SD where d is Cohen’s effect size index, M_1_-M_2_ is the difference between the group means and SD is the standard deviation of either group. The effect sizes were classified to very small (<0.2), small (0.2–0.4), medium (0.5–0.7), large (0.8–1.2) and very large (>1.3)^64^.

### Subcellular Fractionation of DCP-NEt_2_C or DCP-Rho1 Treated Cells and Westen Blot Analysis

A549 cells were cultured in F12K medium supplemented with 10% fetal bovine serum, 100 U/mL penicillin and 100 μg/mL streptomycin at 37 °C in a humidified atmosphere of 5% CO_2_. About 80–85% confluent cells were treated with DCP-NEt_2_C or DCP-Rho1 at 50 μM concentration in F-12K medium (Gibco) without serum for 30 min at 37 °C incubator supplied with 5% CO_2_. After completion of the incubation period, the cells were washed three times with ice-cold DPBS containing 2%FBS and 1% glycine. Subcellular-fractionation was performed using a kit from FOCUS SubCell Kit, G-Biosciences and followed the manufacturer’s protocol with few modifications. Briefly, 300 µL of ice-cold SubCell Buffer-I were added to cells immediately after wash and incubated for 15 min on ice. Cells were transferred to a tube containing 0.5 mm zirconium beads (Sigma-Aldrich) and homogenized using a 15 sec pulse in a beads ruptor (Omni International). Homogenates were transferred to a new microcentrifuge tube and 150 µL of 3X subcellular buffer-II were added to get a 1× final concentration. Cell homogenates were centrifuged at 700 × g for 10 min at 4 °C. Supernatant was transferred to the new centrifuged tube and pellet was collected as the nuclear fraction. Further, nuclear fraction was redissolved in RIPA buffer supplemented with protease and phosphatase inhibitor. The supernatant was centrifuged at 12,000 × g for 15 min at 4 °C to pellet down the mitochondria. The mitochondria containing pellet was resuspended with 50 µL of working mitochondria storage buffer. The supernatant was collected into a new tube and ultracentrifuged at 100,000 × g for 60 min at 4 °C (Beckman TLA-110 rotor). The supernatant containing the soluble cytosolic fraction was collected into a new tube and membraneous pellets were solubilized with 50 µL solubilization buffer supplemented with a protease inhibitor. Equal volumes of subcellular fractions were subjected to 12% SDS-PAGE and the fluorescence was visualized using GE Amersham Imager 600 with the blue channel (Epi Light 460 nm and emission filter 525BP20) for DCP-NEt_2_C and Green channel (Epi Light 530 nm and emission filter 605BP40) for the DCP-Rho1. The gel was stained with Coomassie Brilliant Blue (Thermo Fisher Scientific) to visualize the protein load.

For fractionation control Western blots, the cell fractions were subjected to 12% SDS-PAGE gel and transferred onto a nitrocellulose membrane. After blocking with 5% BSA in TBS-T for 1 hr, the membrane was cut into two parts as indicated by the arrow. The blots were incubated overnight at 4 °C with primary antibodies: Pan-cadherin for the membrane (Santa Cruz Biotechnology, Inc; sc-59876), Calreticulin for the endoplasmic reticulum (Abcam; ab92516), VDAC for the mitochondrial fraction (Cell Signaling, 4866) and PARP for the nuclear fraction (Cell Signaling, 9532). The membranes were washed three times 10 min with TBS-T and secondary antibodies (Anti-mouse IgG, HRP-linked; Cell Signaling 7076 and anti-rabbit, HRP-linked; Cell Signaling 7074 S) were incubated for 1 hr at room temperature. After completion of the incubation period, the membranes were washed three times 10 min with TBS-T and developed with ECL (Amersham). The membranes were stained with reversible protein stain (Thermo Fisher Scientific). Blots were stained with Pierce Reversible stain to visualize membrane-bound protein, the picture was taken under GE Amersham Imager 600.

### Mitochondrial Isolation and Treatment

Mitochondria were isolated from C57Bl/6 mouse liver using a modified procedure according to a published method^65^. Mice were housed in the animal facility of Wake Forest School of Medicine and experiments were conducted in accordance with the National Research Council publication Guide for Care and Use of Laboratory Animals, and approved by Wake Forest Institutional Animal Care and Use Committee. Briefly, 100 mg of liver tissue was weighed and suspended in 1 mL of Chappel Perry (CP) buffer I and minced into small pieces using a sterile pair of scissors. Immediately afterwards the minced tissues were homogenized using an automated homogenizer at a speed of 10,000 rpm for a two second pulse and repeating this five times. This was followed by centrifugation at 600 × g for 10 min and the supernatant was passed through cheese cloth. The filtrate was further washed first with CPII buffer (containing 0.5% fatty acids-free BSA and 0.2 mM ATP) and then CPI buffer (1 mM ATP and no fatty acids-free BSA) by centrifugation at 10,000 g for 10 min.

The isolated mitochondria were suspended in the mitochondrial assay solution (MAS), containing 2 mM malate and varying concentrations of pyruvate (0, 4, 8, 12 or 16 mM) or 1 μM FCCP, and incubated at 37% for 5 min. After this, either TMRE (10 nM) or DCP-NEt_2_C or DCP-Rho1 (both at 10 μM) were added and incubated for 15 min. The mitochondria were centrifuged at 2,000 rpm for 5 min and washed once with MAS. The centrifugation was repeated and the mitochondria were resuspended in MAS for the flow cytometry analysis with the BD Fortessa Cell Analyzer (BD Biosciences). The statistical analysis was done the same way as for the flow cytometry analysis above.

### Silver Nanoparticles Characterization

#### Silver Nanoparticles

Spherical, 5 nm diameter silver nanoparticles (AgNPs) coated with polyvinylpyrrolidone (15–25% silver by mass) were purchased as a dried powder from nanoComposix, Inc (San Diego, CA). Nanoparticles were hydrated in 1 mL of Phosphate Buffered Saline (PBS) (Invitrogen, Carlsbad, California) at a concentration of 20 mg/mL in a 7 mL glass vial. The particles were dispersed by bath sonication. The resulting suspension was opaque and grey-brown in color. For cell culture experiments, the nanoparticles were diluted in cell culture media to the appropriate concentration immediately prior to addition to the wells containing cells.

#### Dynamic Light Scattering

All measurements were made using the Zetasizer Nano ZS90 (Malvern Instruments, UK). The hydrodynamic diameter and ζ-potential of AgNPs was measured at a concentration of 1 mg/mL. Size measurements were taken in PBS (pH 7.4) using a disposable, clear plastic cuvette (Sarstedt, Newton NC) and were optimized using automatic settings. Zeta potential was measured in water (pH 6.5) using disposable folded capillary cells (Malvern Instruments, UK). Each measurement was taken in triplicate.

#### MTT assay

A549 cells were grown in T75 tissue culture flasks in complete media consisting of DMEM/F12 supplemented with 10% FBS, 2 mM L-glutamine, penicillin (250 U/mL), and streptomycin (250 μg/mL) (all from Invitrogen, Carlsbad, California). Cells growing in log phase were trypsinized, washed in PBS, counted using a hemocytometer, and plated on 96-well plates at a density of 5,000 cells per well in 200 μL of complete media. Cells were allowed to recover for 18 h and were then exposed to AgNPs for 72 h. Media containing AgNPs were replaced with 200 µL of media containing 0.5 mg/mL MTT (4,5-dimethylthiazol-2-yl)−2,5-diphenyltetrazolium bromide; Sigma-Aldrich St. Louis, Missouri), and cells were incubated for 1 h at 37 °C. Medium was removed, and cells were lysed in 200 μL of DMSO and absorbance was read using a Molecular Devices Emax Precision Microplate Reader at 560 nm and corrected for background at 650 nm. IC_50_ was calculated using GraphPad Prism software.

## Electronic supplementary material

Supplementary Information
