# Supplementary material for: Mitochondria-targeted Probes for Imaging Protein Sulfenylation
Source: Sci Rep. 2018 Apr 27;8:6635. doi: 10.1038/s41598-018-24493-x (PMC5923234; doi:10.1038/s41598-018-24493-x)

SUPPLEMENTAL INFORMATION

Mitochondria-targeted Probes for Imaging Protein Sulfenylation

Reetta J. Holmila[1], Stephen Vance[2], Xiaofei Chen[1], Hanzhi Wu[1], Kirtikar Shukla[1], Manish S. Bharadwaj[3], Jade Mims[1], Zack Wary[2], Glen Marrs[4], Ravi Singh[5], Anthony J. Molina[3], Leslie, B. Poole[6], S. Bruce King[2], Cristina M. Furdui[1*]

[1] Department of Internal Medicine, Section on Molecular Medicine, Wake Forest University Health Sciences, Winston-Salem, NC 27157, USA

[2] Department of Chemistry, Wake Forest University, Winston-Salem, NC 27109, USA

[3] Department of Internal Medicine, Section on Gerontology and Geriatric Medicine, Wake Forest University Health Sciences, Winston-Salem, NC 27157, USA

[4] Department of Biology, Wake Forest University, Winston-Salem, NC 27109, USA

[5] Department of Cancer Biology, Wake Forest University Health Sciences, Winston-Salem, NC 27157, USA

[6] Department of Biochemistry, Wake Forest University Health Sciences, Winston-Salem, NC 27157, USA

* To whom correspondence should be addressed: Cristina M. Furdui, Department of Internal Medicine, Section on Molecular Medicine, Wake Forest School of Medicine, Medical Center Blvd., Winston-Salem, NC 27157. Tel.: (336) 716-2697; Fax: (336) 716-1214; E-mail: cfurdui@wakehealth.edu.

Supplementary figures

Figure S1. ESI-TOF MS analysis of reduced and oxidized C165A AhpC reaction with Rhodamine B and NEt_2C-N_3. (a) ESI-TOF MS deconvoluted spectra of reduced C165A AhpC reacted for 60 min with NEt_2C-N_3 (2 mM), rhodamine B (2 mM) and MSBT control (5 mM). **(b)** ESI-TOF MS deconvoluted spectra of reduced C165A AhpC reacted with NEt_2C-N_3 (5 mM) to better visualize the adducts species. **(c)** ESI-TOF MS deconvoluted spectra of mixed oxidized C165A AhpC species reacted with NEt_2C-N_3 (5 mM, 60 min), rhodamine B (2 mM, 60 min), and MSBT control (5 mM, 30 min). **(d)** Flow cytometry analysis of A549 cells treated with DCP-Rho1 or rhodamine B (10 μM) (upper panel), and with DCP-NEt_2C or NEt_2C-NH_2 (50 μM) (lower panel) for 30 min and then fixed show increased fluorescence only when labeled with DCP-Rho1 or DCP-NEt_2C.

Figure S2. Subcellular localization of the probes in fixed cells. (a) The A549 cells were treated with DCP-NEt_2C (cyan color) and fixed with methanol; TOMM20 (green) was used as a mitochondrial marker. ICA-plots show colocalization analysis (ICA: intensity correlation analysis). **(b)** Cells were treated with DCP-Rho1 (magenta color) and fixed with methanol; TOMM20 (green) was used as a mitochondrial marker. ICA-plots show colocalization analysis. **(c)** Biotin (magenta color) detected in cells with and without DCP-Bio1 treatment in the absence and presence of tBHP (100 μM), fixed with methanol and imaged. Insets show the imaging with more sensitive settings for the cells without DCP-Bio1 or tBHP treatment.

Figure S3. Subcellular distribution and labeling of oxidized proteins by DCP-NEt_2C and DCP-Rho1 probes using SDS-PAGE and fluorescence imaging. (a) A549 cells were treated with DCP-NEt_2C or DCP-Rho1, processed to isolate subcellular fractions, separated by SDS-PAGE and imaged using fluorescence detection. The C = cytosolic fraction, M = mitochondrial fraction, N = nuclear fraction, and E = membrane fraction. Top panel shows imaging of DCP-NEt_2C and DCP-Rho1 labeled proteins and bottom panel shows total protein staining. **(b)** Corresponding Western blot for fractionation control. The arrow indicates where the membrane was cut to enable simultaneous analysis with antibodies against nuclear, mitochondrial, membrane and ER proteins.

Figure S4. ICA-plots for the colocalization of the probes with mitochondria when the mitochondria membrane potential is disrupted (confocal microscopy images shown in main text **Fig. 4f**). **(a)** A549 cells labeled with DCP-NEt_2C with and without FCCP treatment. **(b)** A549 cells labeled with DCP-Rho1 with and without FCCP treatment.

Figure S5. The effect of disruption of mitochondria membrane potential on mean fluorescence of live cells treated with DCP-NEt_2C or DCP-Rho1.

Figure S6. Western blot analysis of hyperoxidized peroxiredoxins as biomarkers of cellular and

mitochondrial oxidative stress.

Figure S7. Physicochemical characterization and cytotoxicity of AgNPs in A549 cells. Dynamic light scattering (DLS) was used to measure the **(a)** hydrodynamic diameter and **(b)** ζ-potential of AgNPs in PBS (pH 7.4) or water (pH 6.5), respectively. The curves indicate triplicate measurements of each sample. **(c)** A549 cells were exposed to AgNPs for 72 h and cytotoxicity was assessed by MTT assay.

Figure S1

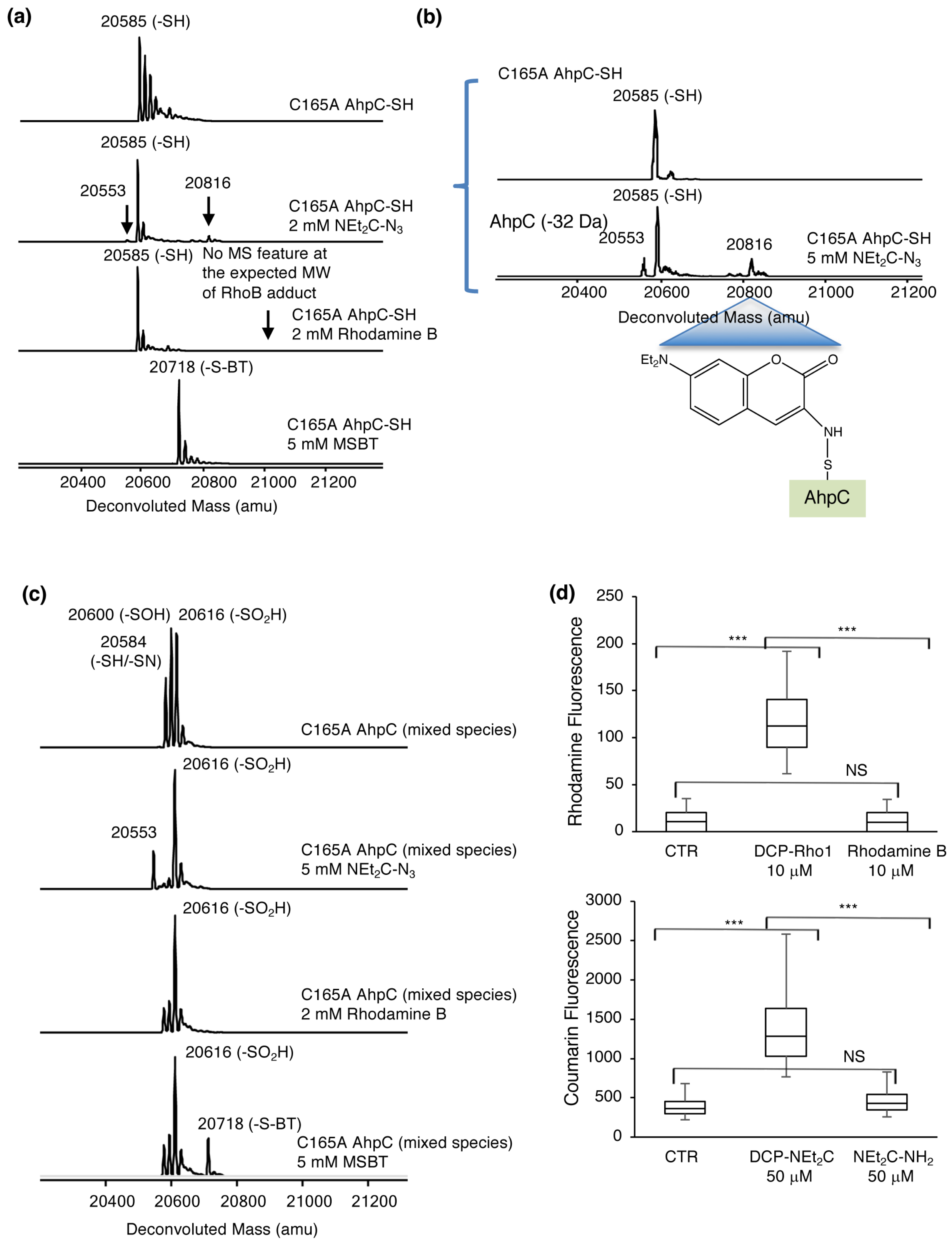

(a)
20585 (-SH)
C165A AhpC-SH
20585 (-SH)
20553
20816
C165A AhpC-SH
2 mM NEt2C-N3
20585 (-SH)
No MS feature at the expected MW of RhoB adduct
C165A AhpC-SH
2 mM Rhodamine B
20718 (-S-BT)
C165A AhpC-SH
5 mM MSBT
20400 20600 20800 21000 21200
Deconvoluted Mass (amu)
(b)
C165A AhpC-SH
20585 (-SH)
20585 (-SH)
AhpC (-32 Da)
20553
20816
C165A AhpC-SH
5 mM NEt2C-N3
20400 20600 20800 21000 21200
Deconvoluted Mass (amu)
Et2N
O
O
NH
S
AhpC
(c)
20600 (-SOH)
20616 (-SO2H)
20584
(-SH/-SN)
C165A AhpC (mixed species)
20616 (-SO2H)
20553
C165A AhpC (mixed species)
5 mM NEt2C-N3
20616 (-SO2H)
C165A AhpC (mixed species)
2 mM Rhodamine B
20616 (-SO2H)
20718 (-S-BT)
C165A AhpC (mixed species)
5 mM MSBT
20400 20600 20800 21000 21200
Deconvoluted Mass (amu)
(d)
Rhodamine Fluorescence
250 200 150 100 50 0
***
***
NS
CTR
DCP-Rho1 10 μM
Rhodamine B 10 μM
Coumarin Fluorescence
3000 2500 2000 1500 1000 500 0
***
***
NS
CTR
DCP-NEt2C 50 μM
NEt2C-NH2 50 μM

Figure S2

(a)

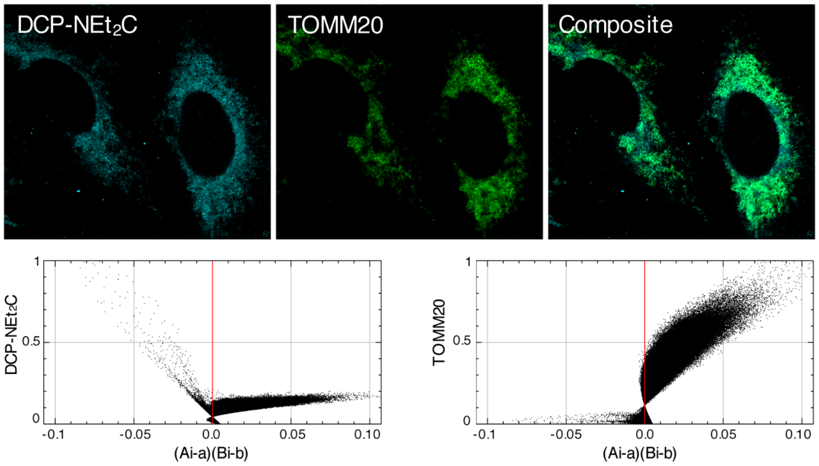

(b)

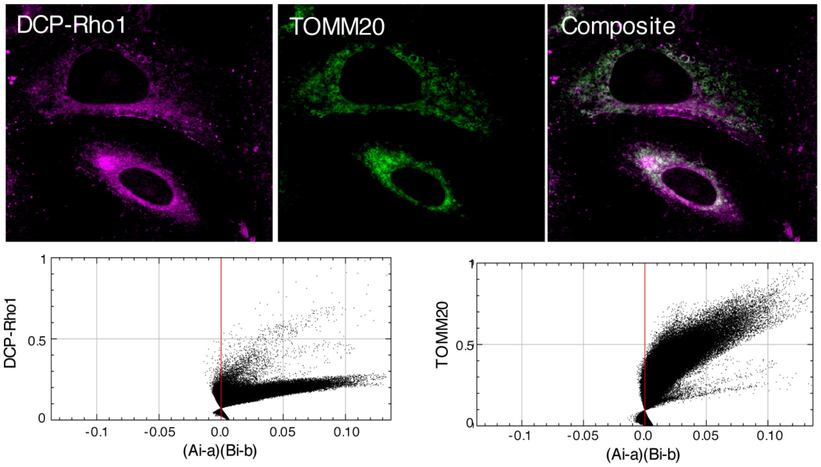

(c)

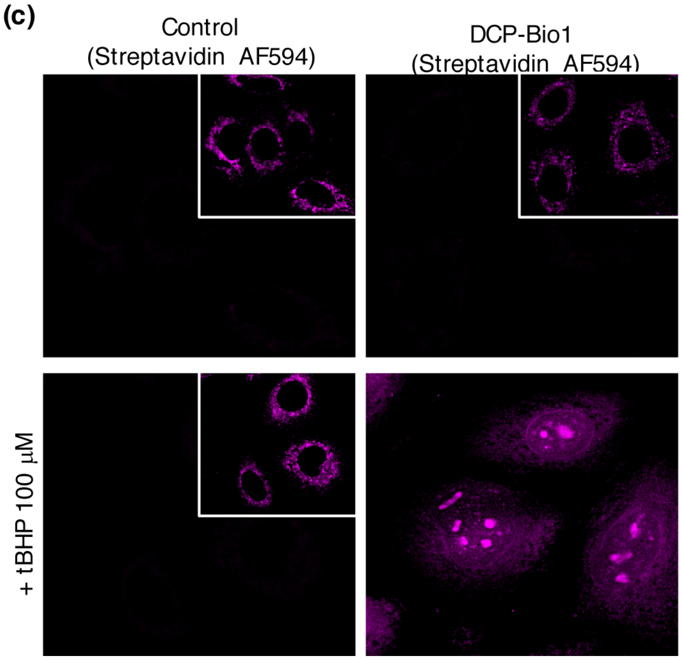

Figure S3

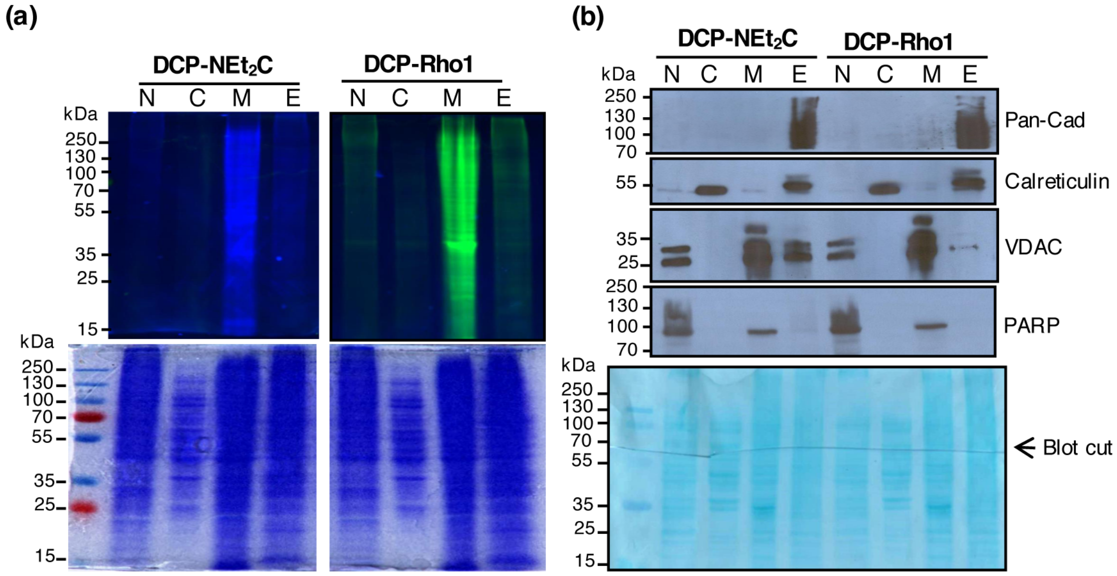

C: Cytosol; N: Nucleus; M: Mitochondria; E: Membrane

Figure S4

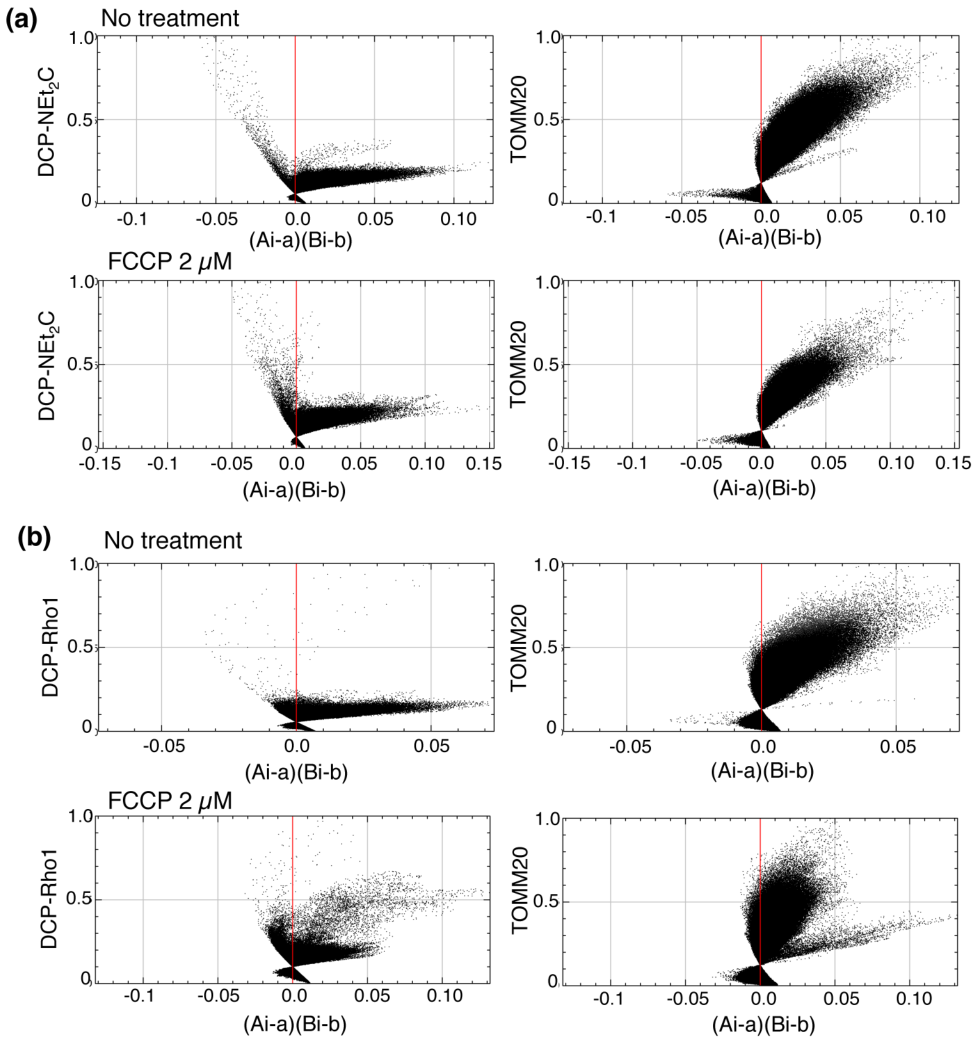

Figure S5

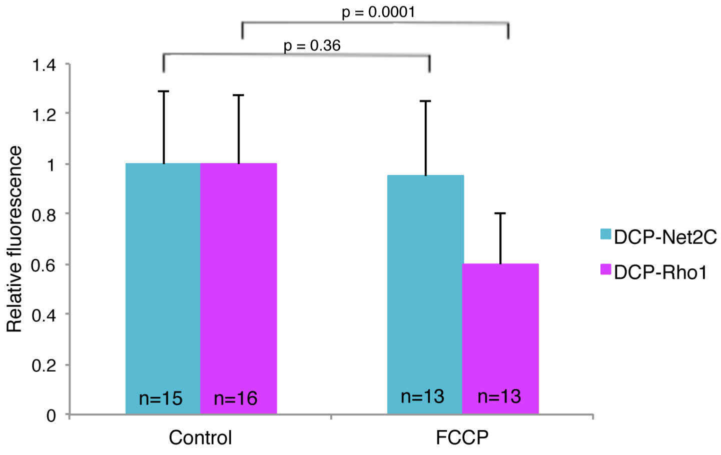

Figure S6

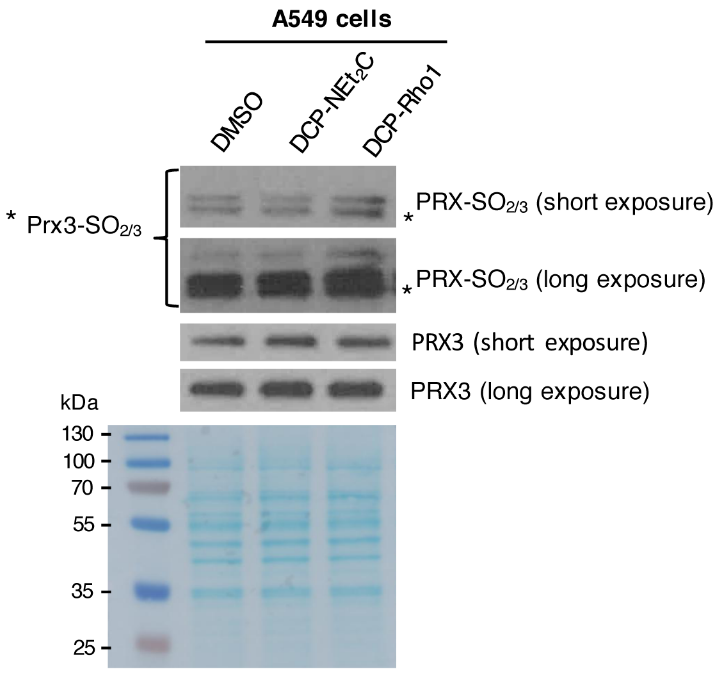

Figure S7

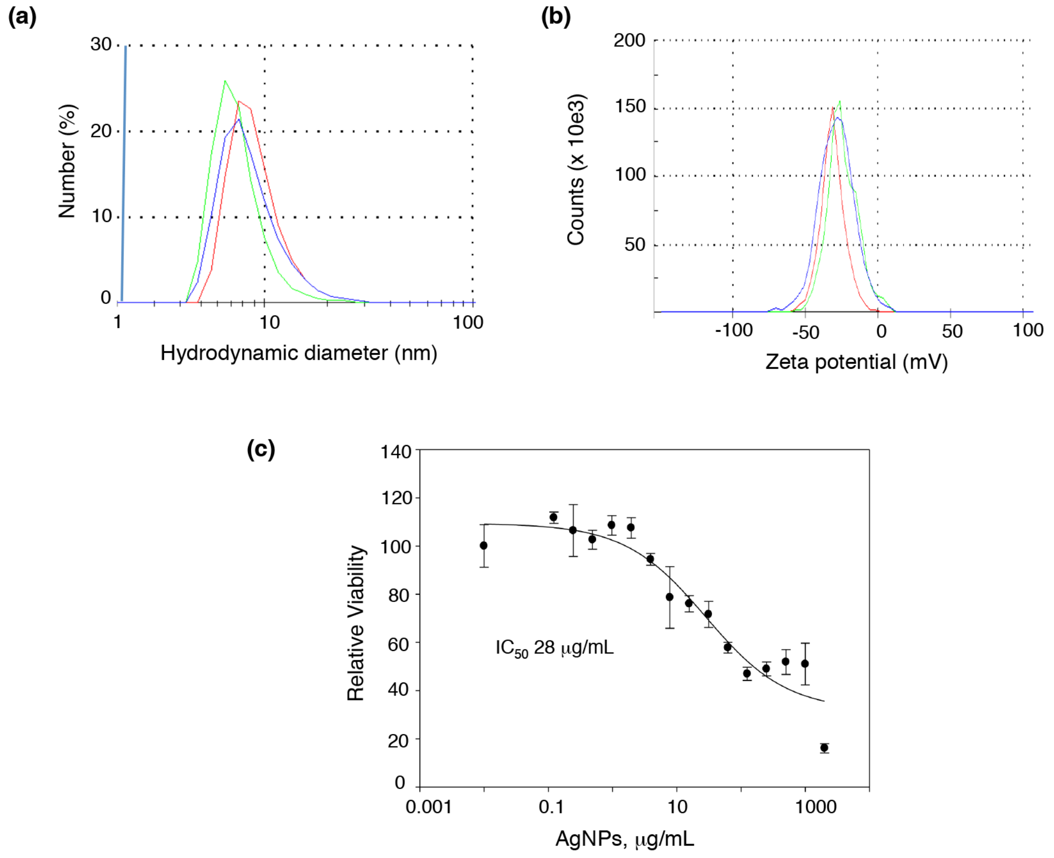

Supplement: Supplementary file 1 — Supplementary Information [file 41598_2018_24493_MOESM1_ESM.pdf]
